# CircRNA/miRNA/mRNA axis participates in the progression of partial bladder outlet obstruction

**DOI:** 10.1186/s12894-022-01132-2

**Published:** 2022-11-25

**Authors:** Baoyi Zhu, Jun Gao, Yuying Zhang, Baojian Liao, Sihua Zhu, Chunling Li, Junhao Liao, Jianjia Liu, Chonghe Jiang, Jianwen Zeng

**Affiliations:** 1 Department of Urology, The Sixth Affiliated Hospital of Guangzhou Medical University (Qingyuan People’s Hospital), B24 Yinquan Road, Qingcheng, Qingyuan, 511500 Guangdong People’s Republic of China; 2 Department of Basic Medical Research, The Sixth Affiliated Hospital of Guangzhou Medical University, Qingyuan, 511518 Guangdong People’s Republic of China; 3 Department of Child Health Care, Shenzhen Longhua Maternity and Child Health Care Hospital, Shenzhen, 518000 Guangdong People’s Republic of China; 4 Guangzhou Regenerative Medicine and Health Guangdong Laboratory, Guangdong Provincial Key Laboratory of Stem Cell and Regenerative Medicine, Guangzhou Institutes of Biomedicine and Health, Chinese Academy of Sciences, Guangzhou, 510700 Guangdong People’s Republic of China

**Keywords:** circRNA/miRNA/mRNA, ceRNA, Bladder outlet obstruction, mRNA sequencing

## Abstract

**Background:**

More and more evidence showed that circRNA/miRNA/mRNA axis played a vital role in the pathogenesis of some diseases. However, the role of circRNA/miRNA/mRNA axis in partial bladder outlet obstruction (pBOO) remains unknown. Our study aimed to explore the complex regulatory mechanism of circRNA/miRNA/mRNA axis in pBOO.

**Methods:**

The pBOO rat model was established, and the bladder tissues were collected for mRNA sequencing. The differentially expressed mRNAs were analyzed by high-throughput sequencing, and the GO and KEGG analysis of the differentially expressed mRNAs were performed. Competing endogenous RNAs (ceRNAs) analysis identified the potential regulation function of circRNA/miRNA/mRNA axis in pBOO. qRT-PCR detected the expression of circRNA/miRNA/mRNA. miRanda software was performed to predict the relationship between circRNA and miRNA, miRNA and mRNA.

**Results:**

Compared with the sham group, a total of 571 mRNAs were differentially expressed in the pBOO group, of which 286 were up-regulated and 285 were down-regulated. GO analysis showed that the mRNAs were mainly involved in cellular process, single-organism process, and cell, etc. KEGG analysis showed that the enriched signaling pathways were metabolic pathways, cell adhesion molecules (CAMs), and HTLV-I infection, etc. Based on the previous transcriptome data and differentially expressed circRNAs, we drew the ceRNA network regulation diagram. qRT-PCR results confirmed that chr3:113195876|113197193/rno-miR-30c-1-3p/Gata4, chr1:126188351|126195625/rno-miR-153-5p/Diaph3, and chr9:81258380|81275269/rno-miR-135b-5p/Pigr axis may have ceRNA function. miRanda confirmed there have the binding sites of circRNA/miRNA/mRNA axis.

**Conclusions:**

CircRNA/miRNA/mRNA axis was involved in the progression of pBOO. Our research on the circRNA/miRNA/mRNA axis revealed new pathogenesis and treatment strategies for pBOO.

## Introduction

Partial bladder outlet obstruction (pBOO) is a common urinary disease, which is commonly seen in clinical benign prostatic hyperplasia (BPH), bladder neck contracture, urethral stricture, congenital urethral malformation and bladder neck tumor, among which BPH is the most common cause of pBOO [1–3]. Studies have shown that pBOO is the initiating factor of the physiological and pathological cascade that leads to deep changes in the structure and function of the bladder [4]. pBOO can trigger inflammatory response in the bladder, accompanied by urodynamic changes of bladder function, leading to organ fibrosis and eventually loss of contractile ability [5]. However, partial pBOO can increase systemic and tissue oxidative stress [6]. In addition to the removal of obstruction by operation, prevention of secondary bladder fibrosis and protection of upper urinary tract function are the key points of pBOO treatment [7]. However, the mechanism of bladder fibrosis after pBOO has not yet been fully clarified at present, and there are still no ideal biomarkers for diagnosis, monitoring and treatment of pBOO. Therefore, there is an urgent need to develop new strategies for the treatment of pBOO.

In the transcriptome, many protein-coding mRNAs and non-protein-coding transcripts are closely related to the pathogenesis of diseases [8, 9]. Circular RNA (circRNA)/microRNA (miRNA)/mRNA axis plays an increasingly vital role in disease progression [10, 11]. CircRNAs were identified as the new star of endogenous non-coding RNAs [12]. CircRNAs competitively bind to miRNA response elements, act as natural miRNA sponges, regulate downstream mRNA expression, and are involved in many human physiology and pathology by competing endogenous RNA (ceRNA) mechanisms [13, 14]. CeRNA forms a large-scale and complex regulatory network in the whole transcriptome, which greatly expands the functional genetic information in the human genome [15]. In addition, RNA sequencing technology has been widely used for effective target screening, especially in biological applications [16]. RNA-sequencing analysis is an open system for discovering new genetic information and has been applied to potential targets of several diseases [17]. Previous studies revealed miRNA may play a role in regulating urothelial permeability and bladder contractility [18, 19]. In view of these data, circRNA/miRNA/mRNA axis may play a crucial part in pBOO, but the regulatory pathway remains unknown.

In the previous study, female Sprague Dawley rats were used to construct a model of pBOO, and then the bladder tissues were collected. High-throughput sequencing was used to analyze the differentially expressed circRNAs in the bladder tissues, and preliminarily determined that circRNAs can participate in the progress of pBOO [20]. In this study, we constructed the pBOO model, and then analyzed the expression profile of mRNAs. The expression profile of mRNAs was analyzed through gene ontology (GO) and Kyoto Encyclopedia of Genes and Genomes (KEGG) to reveal the possible pathogenesis of pBOO. CeRNAs network were established to further elucidate the complex pBOO regulation mechanism. Our findings may provide a new perspective on the pathogenesis and potential diagnosis of pBOO, and contribute to the discovery of potential therapeutic targets for pBOO.

## Material and methods

### Animal

Healthy female Sprague Dawley rats, aged 10 weeks, weighing 200–250 g, were provided by the Southern Medical University (Guangzhou, China). The 16 SD rats were randomly divided into sham and pBOO groups with 8 rats in each group. During the experiment, the animals grew under natural conditions, with the temperature set to 21–24 °C and humidity at 50–70%. The study was carried out in compliance with the ARRIVE guidelines. This animal experiment was approved by Experimental Animal Care and Usage Committee of the Six Affiliated Hospital of Guangzhou Medical University (Qingyuan People’s Hospital) (Qingyuan, China).

### Establishment of the pBOO model

According to the previous studies, we built pBOO rats model [21, 22]. In short, rats expose the bladder and proximal urethra through an incision in the lower abdomen under anesthesia with isoflurane. Through careful observation, the proximal urethra was away from the vaginal wall to avoid damage to the peripheral blood vessels. A metal rod with a diameter of 1 mm is placed next to the proximal urethra, and 4–0 polypropylene ligature is tied around the urethra and the metal rod to cause obstruction. The metal rod was then removed and the abdominal incision was closed. In the sham group, following the same procedure, the suture was loosely tied around the urethra without obstruction. Ten days after surgery, bladder tissue was taken from these rats for mRNAs sequencing.

### High-throughput sequencing

Total RNA from bladder tissues was extracted with the TRIzol™ reagent (Invitrogen, CA, USA) according to the manufacturer’s instructions. The Ribo-Zero Gold RNA removal Kit (Illumina, San Diego, CA, USA) was used to remove the ribosomal RNA. The rRNA-depleted RNAs were further incubated at 37 °C for 1 h with 10 U/μg RNase R (Thermo Fisher Scientific). The remaining RNAs were used to construct cDNA libraries, according to the protocol of the mRNA-Seq sample preparation kit (Illumina, San Diego, CA, USA). The sequencing instrument used was Illumina Hiseq2500 platform, and the sequencing mode used was PEl50.

### Data analysis

The average sequencing depth of six samples was 6.41G.The quality of sequenced reads was controled by FastQC (v0.11.3) [23] and the clean reads were mapped to the rat reference genome (Rn6) by HISAT2 (2.1.0) [24]. The DESeq2 package (1.32.0) in R 3.3.2 software was also used to process quantile normalization and analyze the differentially expressed mRNAs. The mRNAs with |log_2_fold change|≥ 1 and *P* < 0.05 were considered as differentially expressed. The expression patterns of the mRNAs among the samples were obtained using hierarchical clustering.

### GO and KEGG enrichment analysis

GO enrichment analyses of differentially expressed mRNAs between the sham and pBOO groups were conducted using the Bioinformatics Tool (DAVID, version 6.8, https://david.ncifcrf.gov/) [25, 26]. KEGG pathway enrichment analyses were performed using the KOBAS 2.0 software to clarify differentially expressed mRNAs-related signaling pathways [27].

### Construction of the ceRNA network

With the deepening of transcriptome research, miRNA response elements have been found to exist on circRNA, which means that the same miRNA can bind to multiple types of RNA and form a competitive relationship with different RNA molecules bound with the same miRNA [15]. The differentially expressed circRNAs were determined based on previous studies [20]. Combined with differentially expressed mRNAs (|log_2_fold change| ≥ 1 and *P* < 0.05) and differentially expressed circRNAs (|log_2_fold change| ≥ 1 and *P* < 0.05), a potential ceRNA network was constructed by base pairing and integrating the predicted results. In order to explore circRNA/miRNA/mRNA role in the pathogenesis of pBOO, Cytoscape software (version 3.7.2) was used to visualize the results.

### Quantitative real-time PCR (qRT-PCR)

qRT-PCR was applied to test the relative expression levels of chr3:113195876|113197193, rno-miR-30c-1-3p, Gata4, chr5:122655270|122671094, rno-miR-185-3p, chr1:126188351|126195625, rno-miR-153-5p, Diaph3, chr9:81258380|81275269, rno-miR-135b-5p, and Pigr in bladder tissues. In short, total RNA was extracted by Trizol method, RNA was reversely transcribed into cDNAs in accordance with the instruction of a Reverse transcription kit (CW2569, CWBIO, China). The relative expression of genes was tested by SYBR Green qPCR mix (Invitrogen) on ABI 7900 system. The internal reference gene was GAPDH and U6, and 2^−ΔΔCt^ method was used to calculate the relative transcription level of the target gene. The primer sequences used in this study are showed in Table 1.

**Table 1:** The primer sequences used in this study

Primer name	Sequence (5′-3′)
R-Gata4-F	CTGCGAGACACCCCAATCTC
R-Gata4-R	GCCGGTTGATACCATTCATCTTG
R-Pigr-F	TGCTGGACTGTATGTTTGCC
R-Pigr-R	GCATCATTTGCCACTTCACG
R-Diaph3-F	CCGGGTGCCTTATGAGAAAATC
R-Diaph3-R	TGCTCATCACGACAGCAAAC
rno-miR-30c-1-3p-RT	GTCGTATCCAGTGCAGGGTCCGAGGTATTCGCACTGGATACGACGGAGTA
rno-miR-30c-1-3p-F	CTGGGAGAGGGTTGTTTA
rno-miR-153-5p-RT	GTCGTATCCAGTGCAGGGTCCGAGGTATTCGCACTGGATACGACAGCTGC
rno-miR-153-5p-F	GTCATTTTTGTGATGTTGC
rno-miR-135b-5p-RT	GTCGTATCCAGTGCAGGGTCCGAGGTATTCGCACTGGATACGACTCACAT
rno-miR-135b-5p-F	TATGGCTTTTCATTCCTAT
Universe-R	GTGCAGGGTCCGAGGT
R-chr3:113195876|113197193-F	TGGTTCACTCTCTGACGTCT
R-chr3:113195876|113197193-R	TGCAAAGGTGGCTGATGACA
R-chr5:122655270|122671094-F	AGGAAATCATGGTAGGCTCCA
R-chr5:122655270|122671094-R	GCATTCAGAAGAAAAGCAAGTCAG
R-chr1:126188351|126195625-F	TCCTTTTAGCACATCAGAAATCACT
R-chr1:126188351|126195625-R	GCTCCCATGACAGGCCTC
Rat-GAPDH-F	GCAAGAGAGAGGCCCTCAG
Rat-GAPDH-R	TGTGAGGGAGATGCTCAGTG
Rat-U6-F	CTCGCTTCGGCAGCACA
Rat-U6-R	AACGCTTCACGAATTTGCGT

### Bioinformatics analysis

We used miRanda (version 0.10.80) software to predict the relationship between circRNAs and miRNAs, miRNAs and mRNAs. The binding sites of chr3:113195876|113197193 and rno-miR-30c-1-3p, rno-miR-30c-1-3p and Gata4, chr1:126188351|126195625 and rno-miR-153-5p, rno-miR-153-5p and Diaph3, chr5:122655270|122671094 and rno-miR-185-3p, rno-miR-185-3p and Pigr were detected.

### Statistical analysis

Data were presented as the mean ± standard deviation (SD) and evaluated by the Graphpad 8.0 software. All data were firstly subjected to Shapiro–Wilk normality test. 2 tailed unpaired Student’s T-test or one-way ANOVA test shall be conducted for data with normal distribution, and Mann Whitney test or Kruskal Wallis ANOVA test shall be conducted for data with non normal distribution. *P* < 0.05 was considered as statistically significant.

## Results

### High-throughput sequencing analysis of differentially expressed mRNAs

In order to further explore the mechanism of circRNA/miRNA/mRNA regulation of pBOO, we used high-throughput sequencing to analyze the differentially expressed mRNAs in the bladder tissues.

**Fig. 1:**
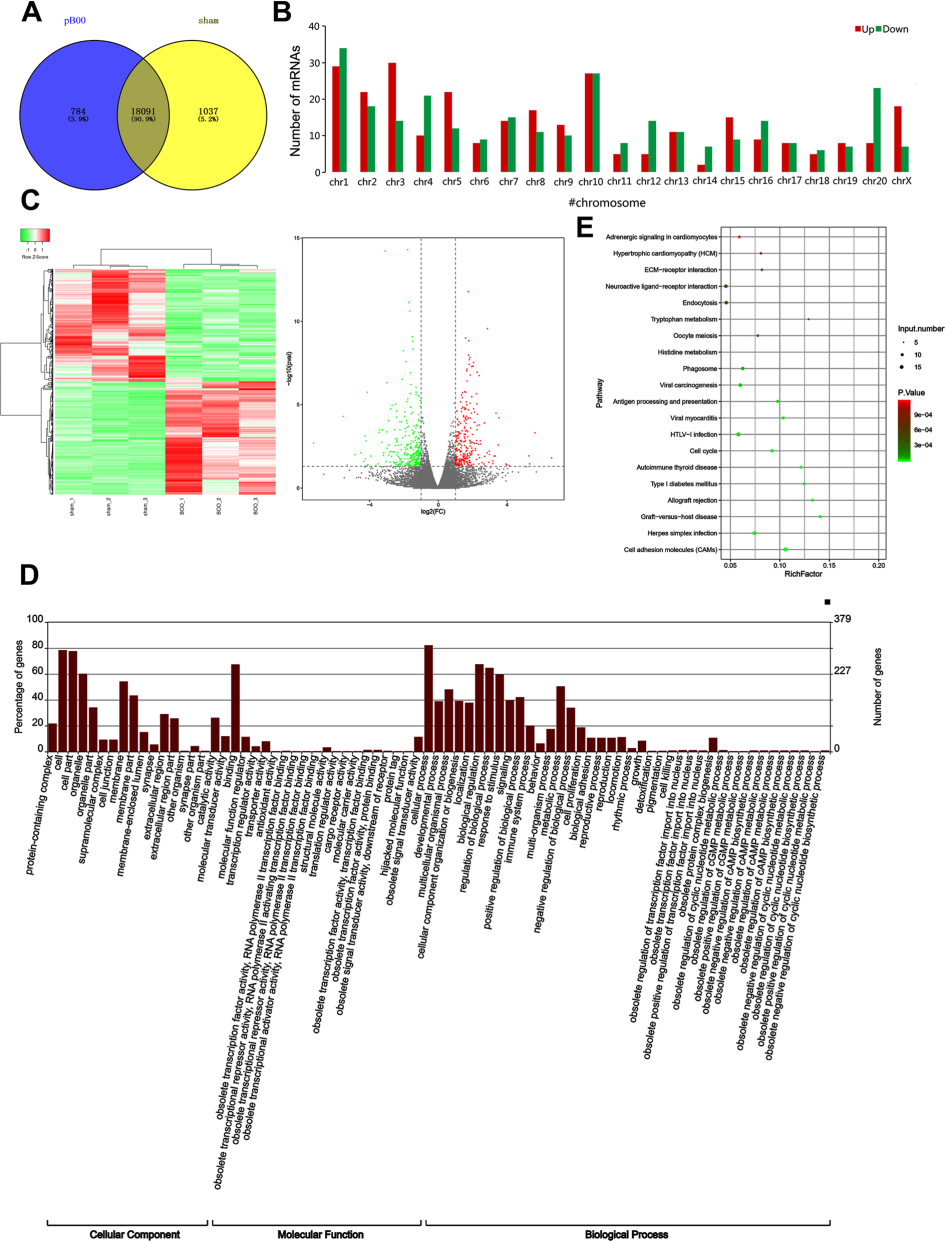
High-throughput sequencing analysis of differentially expressed mRNAs. **A** Venn diagrams of mRNAs were identified in sham and pBOO groups. **B** Number of differential expression mRNAs from different chromosomes. Green and red columns represent numbers of down-regulated and up-regulated mRNAs in pBOO. **C** Heat map (left) and volcano map (right) of differentially expressed mRNAs. **D** GO analysis diagram of differentially expressed mRNAs. **E** KEGG analysis was used to determine the top 20 enrichment pathways

As shown in Fig. 1A, there are 18,091 mRNAs commonly expressed in the sham and pBOO groups. In addition, 784 mRNAs were unique to the sham group and 1037 mRNAs were unique to the pBOO group. Figure 1B showed the number of mRNAs differentially expressed on different chromosomes. The green and red columns represent the number of down-regulated and up-regulated mRNAs in pBOO, respectively. Hierarchical clustering analysis was first used to reveal the expression profiles of 571 differentially expressed mRNAs (|log_2_fold change| ≥ 1 and *P* < 0.05) between the sham and pBOO groups, of which 286 were up-regulated and 285 were down-regulated (Fig. 1C). GO analysis clarified functional annotations including biological processes (BP), cellular components (CC), and molecular functions (MF) (Fig. 1D). CC reflects that differentially expressed mRNA is distributed in different cell components. MF showed that mRNA related to binding, catalytic activity and molecular transducer activity was the most common. BP shows that cellular process is the most disturbed biological process. Next, KEGG analysis was used to determine the top 20 enrichment pathways. The signaling pathways that changed in the sham, pBOO groups were: metabolic pathways, cell adhesion molecules (CAMS), and HTLV-I infection, etc. (Fig. 1E). Therefore, based on the above results, we were able to successfully screen the mRNAs in pBOO rats.

### The ceRNA network regulation diagram of circRNA/miRNA/mRNA

**Fig. 2:**
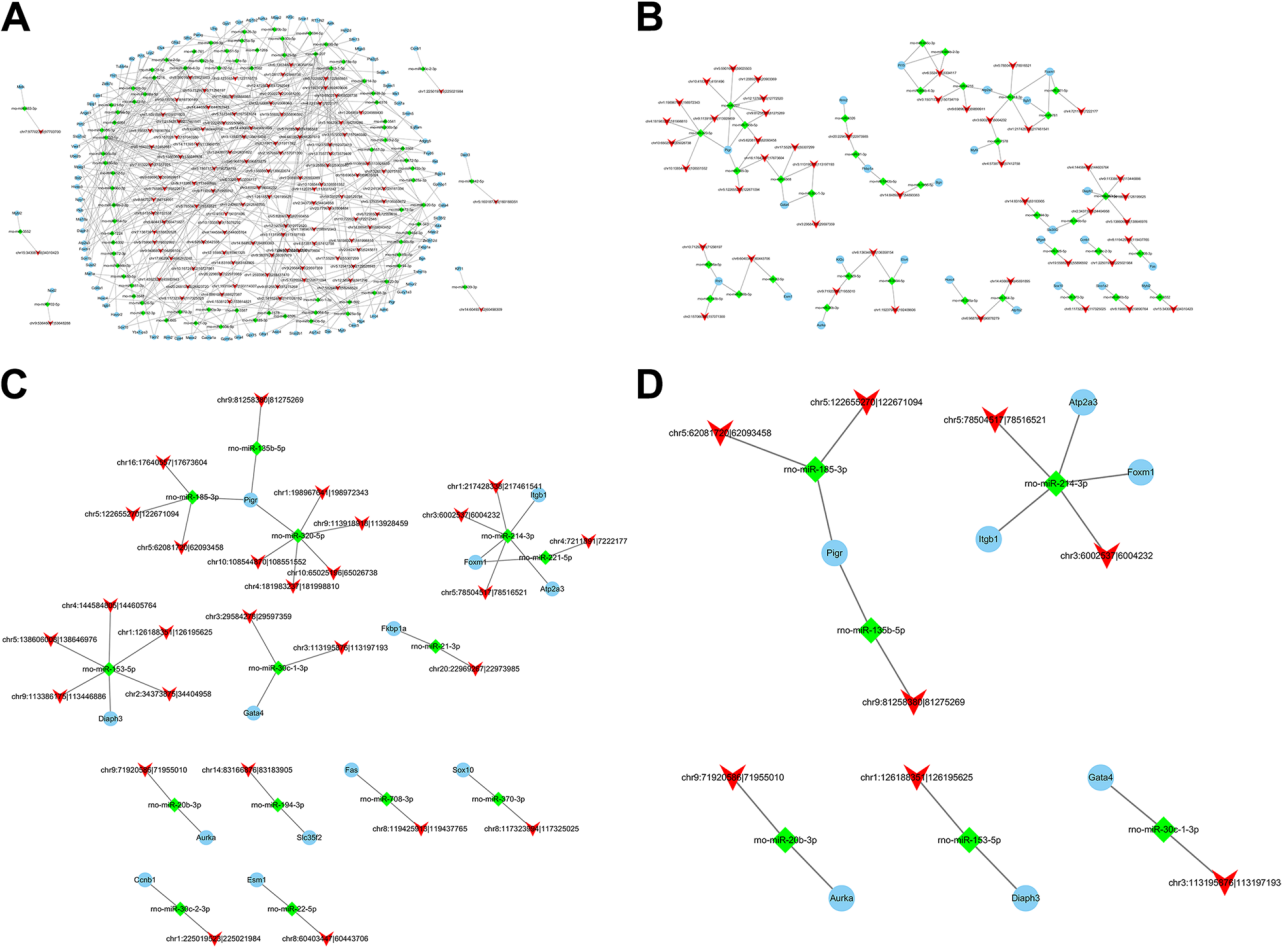
The ceRNA network regulation diagram of circRNA/miRNA/mRNA. **A** The ceRNA total network regulation diagram of circRNA/miRNA/mRNA. **B** The network diagram of ceRNA that target genes are related to bladder function, and numTargetsPer100 bp are greater than 0.2. **C** and **D** The network diagram of ceRNA that the miRNAs and circRNAs origin genes are related to bladder function, respectively

Based on the previous transcriptome data and differentially expressed circRNAs, we combined with the differentially expressed mRNAs (|log_2_fold change| ≥ 1 and *P* < 0.05, a total of 571) and differentially expressed circRNAs (|log_2_fold change| ≥ 1 and *P* < 0.05, a total of 3051) to make ceRNA mechanism form. The ceRNA general network regulation diagram of circRNA/miRNA/mRNA was drawn according to the increase or decrease of circRNA and mRNA expression, and the miRNA binding sites in circRNAs and mRNA were more than 3 (Fig. 2A). Among them, there were 38 circRNAs with up-regulated expression and 79 circRNAs with down-regulated expression. Then we screened target genes that are related to bladder function, and numTargetsPer100 bp are greater than 0.2, and draw a ceRNA network diagram (Fig. 2B). Among them, there are 22 circRNAs with up-regulated expression and 24 circRNAs with down-regulated expression. Next, we screened the miRNAs related to bladder function and drew ceRNA network diagram. Among them, there were 14 circRNAs with up-regulated expression and 13 circRNAs with down-regulated expression (Fig. 2C). Finally, in the filter result of Fig. 2B, we further screened the circRNAs origin genes related to bladder function and drew ceRNA network diagram. There were 3 circRNAs with up-regulated expression and 5 circRNAs with down-regulated expression (Fig. 2D). Therefore, next we verified the circRNA/miRNA/mRNA in pBOO rats.

### The identification of the screened circRNA/miRNA/mRNA axis

**Fig. 3:**
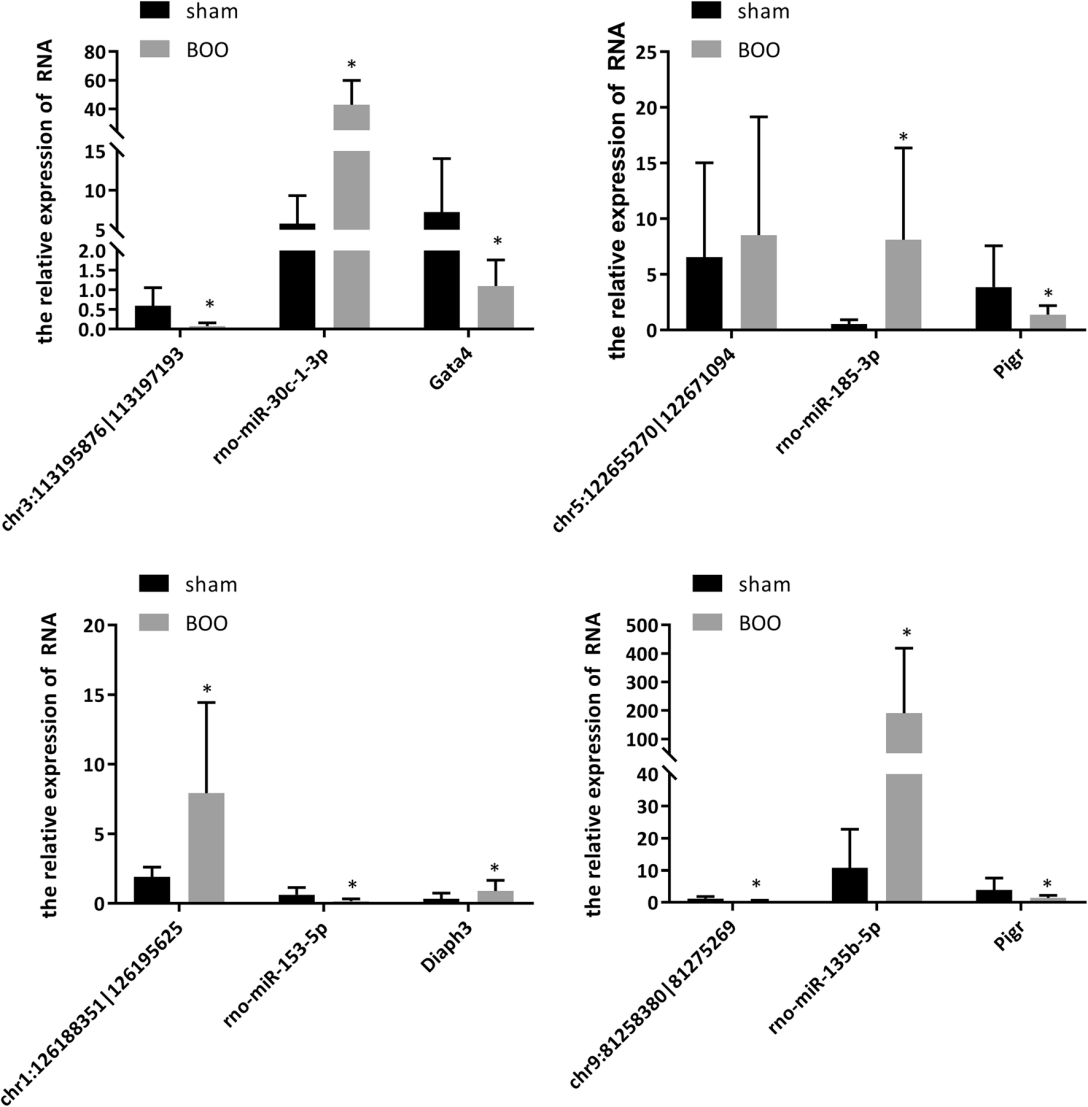
qRT-PCR was used to detect the expression of circRNA/miRNA/mRNA. *P < 0.05 VS sham group

Next, we used qRT-PCR to detect the expression of circRNA/miRNA/mRNA to explore the mechanism of pBOO. The results showed that, compared with the sham group, chr5:122655270|122671094, chr1:126188351|126195625, rno-miR-30c-1-3p, rno-miR-185-3p, rno-miR-135b-5p, and Diaph3 were up-regulated in pBOO group, while chr3:113195876|113197193, chr9:81258380|81275269, rno-miR-153-5p, Gata4, and Pigr were down-regulated (Fig. 3). Among them, chr3:113195876|113197193/rno-miR-30c-1-3p/Gata4, chr1:126188351|126195625/rno-miR-153-5p/Diaph3, and chr9:81258380|81275269/rno-miR-135b-5p/Pigr axis might have ceRNA function. It is worth noting that the expression of rno-miR-135b-5p in BOO group is about 19 times that of sham group, which is more obvious than other molecules, suggesting that this molecule may play a more important role in the regulation of BOO process.

### miRanda analyzed the binding sites of circRNA/miRNA/mRNA axis

**Fig. 4:**
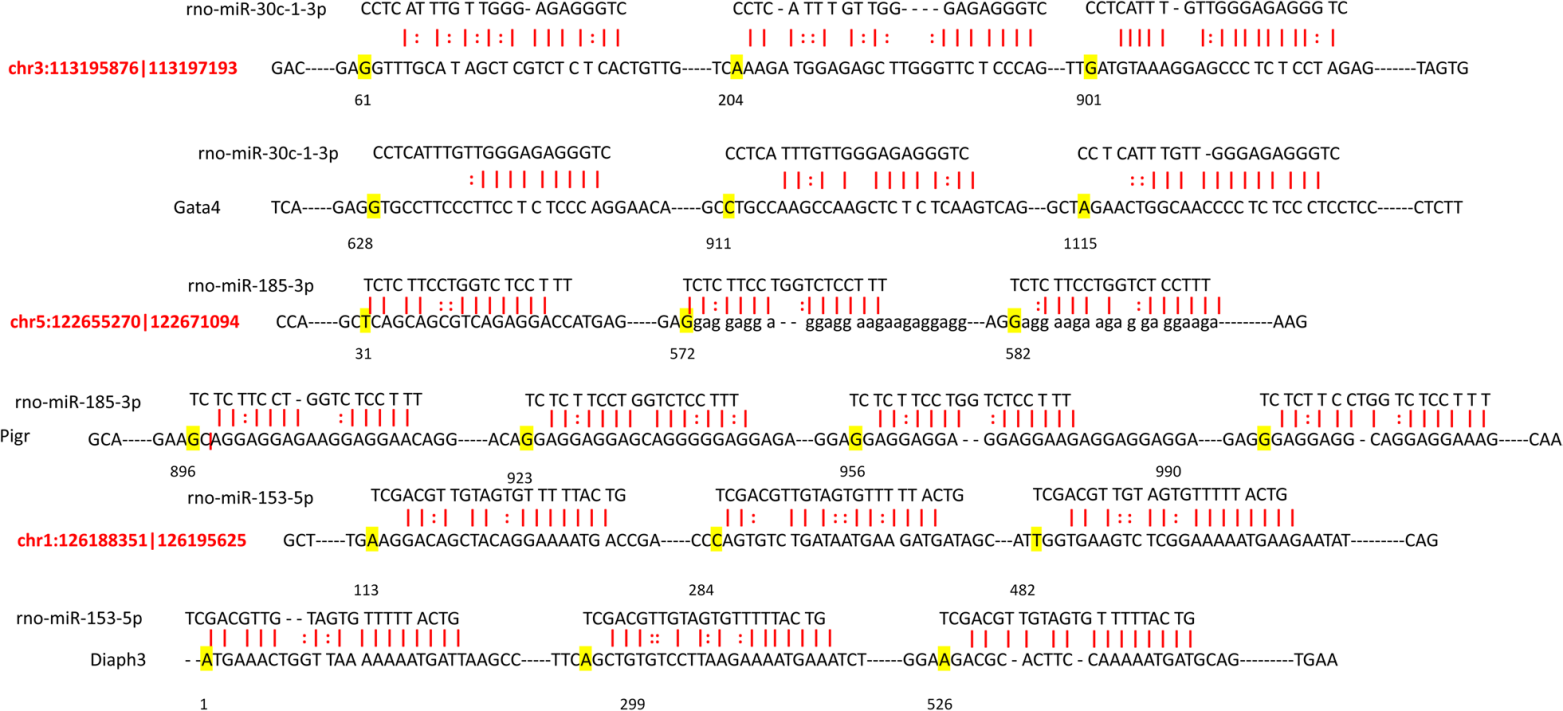
miRanda analyzed the binding sites of circRNA/miRNA/mRNA axis

Further we used miRanda to analyze the circRNA/miRNA/mRNA axis that was consistent with the expression expectation. As shown in Fig. 4, There were binding sites between chr3:113195876|113197193 and rno-miR-30c-1-3p, rno-miR-30c-1-3p and Gata4, chr1:126188351|126195625 and rno-miR-153-5p, rno-miR-153-5p and Diaph3, chr5:122655270|122671094 and rno-miR-185-3p, rno-miR-185-3p and Pigr. Based on the results of qRT-PCR and bioinformatics analysis, we found that chr3:113195876|113197193/rno-miR-30c-1-3p/Gata4, chr1:126188351|126195625/rno-miR-153-5p/Diaph3 axis may participate in the progress of pBOO. Although we confirmed the circRNA/miRNA/mRNA axis in pBOO, whether the circRNA/miRNA/mRNA axises are involved in pBOO requires further investigation.

## Discussion

pBOO occurs in more than 20% of adults and may lead to changes in bladder structure and function [28]. However, pBOO mechanism is still unclear. Studies have shown that circRNAs can act as "miRNA sponge", and the vital regulatory role of circRNAs in miRNA has attracted increasing attention [29]. Few studies have been conducted on mRNAs profile and circRNA-miRNA-mRNA network in the mechanism of pBOO. In order to reveal the biological function of circRNA-miRNA-mRNA and discover new transcripts in pBOO, we performed high-throughput sequencing and bioinformatics studies in the pBOO model to fill in the gaps.

It is well known that mRNA degradation plays a critical part in the post-transcriptional regulation after gene expression. miRNAs are key elements in mRNA degradation and ceRNAs [30]. CeRNA has a significant influence on the formation and progression of cancers by regulating mRNA expression. The discovery of circRNAs add new complexity to the regulation of gene expression mainly through epigenetic control of circRNA-miRNA-mRNA axis [31]. It has been reported that many regulatory miRNAs are involved in bladder pathology and function [32, 33]. Various miRNAs have been shown to be related to the bladder remodeling process caused by pBOO [34]. In contractile bladders from pBOO patients, the down-regulation of miR-199a-5p promotes the development of the disease [35]. In this research, we constructed the circRNA-miRNA-mRNA ceRNA network to study its role in pBOO. Our co-expression network is the primary way to predict the function of circRNA-miRNA-mRNA.

To better explain the effect of significantly dysregulated mRNA expression, we screened the miRNAs and circRNAs origin genes associated with bladder function. From the ceRNA network, we can conclude that circRNAs with different expressions are involved in pBOO through sponge-related miRNAs. Therefore, genes involved in the ceRNA network can help us understand the mechanism of the occurrence and development of pBOO. Combined with clinical data, these RNAs also have potential biomarkers for the diagnosis and prognosis of pBOO. In order to systematically and comprehensively understand the potential function of mRNA in pBOO, we conducted GO and KEGG pathway analysis of 571 differentially expressed mRNAs. Based on our sequencing results, we found that differentially regulated mRNAs were significantly enriched in cellular process, single-organism process, cell etc. KEGG pathway analysis showed that these differentially expressed mRNAs are involved in metabolic pathways, cell adhesion molecules (CAMs), and HTLV-I infection, etc. However, these interesting findings should be further confirmed in future studies.

In the ceRNA network, we selected four circRNA-miRNA-mRNAs, chr3:113195876|113197193/rno-miR-30c-1-3p/Gata4, chr5:122655270|122671094/rno-miR-185-3p/Pigr, chr1:126188351|126195625/rno-miR-153-5p/Diaph3, and chr9:81258380|81275269/rno-miR-135b-5p/Pigr, and qRT-PCR was performed to detect their expression. Compared with the sham group, the expression of these detected RNAs is different in pBOO group. Previous studies found that miR-30c-1-3p inhibited the resistance of prostate cancer to androgen ablation therapy by targeting androgen receptor variant 7 [36]. CircRNA_000203 aggravates cardiac hypertrophy by inhibiting the binding of miR-26b-5p and miR-140-3p to Gata4 [37]. Our results suggest that chr3:113195876|113197193 and Gata4 were down-regulated, while rno-miR-30c-1-3p was up-regulated in pBOO group. Hsa_circ_0088233 attenuate the proliferation, migration and invasion of prostate cancer cells by targeting hsa-miR-185-3p [38]. Polymerized immunoglobulin receptor (PIGR) plays an oncogenic role in hepatocellular carcinoma through activation of ribosomal pathways [39]. We found that chr5:122655270|122671094, rno-miR-185-3p were up-regulated, while Pigr was down-regulated in pBOO group. This showed chr5:122655270|122671094/rno-miR-185-3p/Pigr may not be through ceRNA mechanisms regulating pBOO.

It has been reported that miR-153-5p promotes the sensitivity of colorectal cancer cells to oxaliplatin through targeting Bcl-2 mediated autophagy pathway [40]. The gene encoding the cytoskeleton modulator DIAPH3 is lost frequently in metastatic prostate cancer, and DIAPH3 silencing induces a shift to an amoeba-like tumor phenotype in a variety of cellular settings. It is accompanied by an increase in tumor cell migration, invasion and metastasis [41]. Our research showed that chr1:126188351|126195625, Diaph3 were up-regulated, while rno-miR-153-5p was down-regulated in pBOO group. MiR-135b-5p has been found to play an important role in ovarian cancer, gastric cancer and other diseases [42, 43]. We found that chr9:81258380|81275269, Pigr were down-regulated, while rno-miR-135b-5p was up-regulated in pBOO group. There results suggest that chr3:113195876|113197193/rno-miR-30c-1-3p/Gata4, chr1:126188351|126195625/ rno-miR-153-5p/Diaph3 axis may play a significant role in the pathogenesis of pBOO, and they may be new molecular biomarkers of pBOO. However, this study still has some limitations and further investigation is needed to verify the exact role of the identified circRNA/miRNA/mRNA axis in the pathogenesis of pBOO.

## Conclusion

Based on high-throughput sequencing and bioinformatics analysis, we found chr3:113195876|113197193/rno-miR-30c-1-3p/Gata4, chr1:126188351|126195625/rno-miR-153-5p/Diaph3 axis may be involved in the progress of the pBOO. This may be valuable for the diagnosis and treatment of pBOO and may lead to the discovery of new potential biomarkers. Further study of the functions of these RNAs will help us to understand the mechanism and progression of pBOO.

## Data Availability

The datasets generated and/or analysed during the current study are available in online repositories. The circRNA sequencing data are available via SRA data (BioProject Accession Number: PRJNA772547).

## Abbreviations

pBOO: Partial bladder outlet obstruction
BPH: Benign prostatic hyperplasia
CAMs: Cell adhesion molecules
circRNA: Circular RNA
miRNA: MicroRNA
ceRNA: Competing endogenous RNA
GO: Gene ontology
KEGG: Kyoto Encyclopedia of Genes and Genomes
qRT-PCR: Quantitative Real-time PCR
SD: Standard deviation
ANOVA: Analysis of variance
BP: Biological processes
CC: Cellular components
MF: Molecular functions
PIGR: Polymerized immunoglobulin receptor
